# Racial and Ethnic Differences in Rural-Urban Trends in 5-Year Survival of Patients With Lung, Prostate, Breast, and Colorectal Cancers: 1975-2011 Surveillance, Epidemiology, and End Results (SEER)

**DOI:** 10.1001/jamanetworkopen.2022.12246

**Published:** 2022-05-19

**Authors:** Marquita W. Lewis-Thames, Marvin E. Langston, Saira Khan, Yunan Han, Lindsay Fuzzell, Shuai Xu, Justin Xavier Moore

**Affiliations:** 1 Department of Medical Social Science, Center for Community Health, Feinberg School of Medicine, Northwestern University, Chicago, Illinois; 2 Division of Research, Kaiser Permanente, Northern California, Oakland, California; 3 Department of Epidemiology and Population Health, Stanford University, Stanford, California; 4 Epidemiology Program, College of Health Sciences, University of Delaware, Newark; 5 Division of Public Health Sciences, Department of Surgery, Washington University School of Medicine, St Louis, Missouri; 6 Department of Health Outcomes and Behavior, H. Lee Moffitt Cancer Center, Tampa, Florida; 7 Cancer Prevention, Control, and Population Health Program, Department of Medicine, Augusta University, Augusta, Georgia; 8 Institute of Preventive and Public Health, Augusta University, Augusta, Georgia

## Abstract

**Importance:**

Considering reported rural-urban cancer incidence and mortality trends, rural-urban cancer survival trends are important for providing a comprehensive description of cancer burden. Furthermore, little is known about rural-urban differences in survival trends by racial and ethnic groups.

**Objective:**

To examine national rural-urban trends in 5-year cancer-specific survival probabilities for lung, prostate, breast, and colorectal cancers in a diverse sample of racial and ethnic groups.

**Design, Setting, and Participants:**

This cross-sectional study used an epidemiologic assessment with 1975 to 2016 Surveillance, Epidemiology, and End Results (SEER) data to analyze patients diagnosed no later than 2011. Patients were classified as living in rural and urban counties based on the 2013 Rural-Urban Continuum Codes.

**Main Outcomes and Measures:**

The 5-year cancer-specific survival probability of urban and rural patients for each cancer type was estimated by fitting Cox proportional hazard regression models accounting for race, ethnicity, tumor characteristics, and other sociodemographic characteristics. A generalized linear regression model was used to estimate the mean estimated probability of survival for each stratum. Joinpoint regression analysis estimated periods of significant change in survival.

**Results:**

In this study, data from 3 659 417 patients with cancer (median [IQR] age, 67 [58-76]; 1 918 609 [52.4%] male; 237 815 [6.5%] Hispanic patients; 396 790 [10.8%] Black patients; 2 825 037 [77.2%] White patients) were analyzed, including 888 338 patients with lung cancer (24.3%), 750 704 patients with colorectal cancer (20.5%), 987 826 patients with breast cancer (27.0%) breast, and 1 023 549 patients with prostate cancer (28.0%). There were 430 353 rural patients (11.8%). Overall, there was an equal representation of rural and urban men. Rural patients were likely to be non-Hispanic White individuals, have more cases of distant tumors, and be older. Rural and non-Hispanic Black patients for all cancer types often had shorter survival. From 1975 to 2016, the 5-year lung cancer survival rate was shorter for non-Hispanic Black rural patients in 1975 at 48%, while increasing to 57% for both non-Hispanic Black urban and rural patients in 2011, but still the shortest among all cancer types. In 1975, the longest survival rate was observed in urban Asian and Pacific Islander patients with breast cancer at 86%, and in 2011, the longest survival rate was observed in urban non-Hispanic White patients with XX cancer at 92%.

**Conclusions and Relevance:**

Even after accounting for sociodemographic and tumor characteristics, these findings suggest that non-Hispanic Black patients with cancer are particularly vulnerable to cancer burden, and resources are urgently needed to reverse decades-old survival trends.

## Key Points

**Question:**

What are the rural-urban trends in overall and cancer-specific survival across the most prevalent cancer types (ie, lung, prostate, breast, and colorectal cancers)?

**Findings:**

This cross-sectional study of 3 659 417 patients with cancer used an epidemiological assessment of 1975 to 2011 data from the SEER database and found that 5-year cancer-specific survival trends were often lower for rural and non-Hispanic Black patients.

**Meaning:**

These findings suggest that the survival of rural patients has trailed the survival of urban patients for almost 40 years, regardless of sociodemographic and clinical variables.

## Teaser

This cross-sectional study uses an epidemiological assessment to examine rural-urban trends in the 5-year survival of patients with lung, prostate, breast, and colorectal cancers.

## Introduction

For many cancer types, cancer incidence and mortality disproportionately affect rural populations.^1,2^ When compared with residents in urban areas, individuals in rural areas have an increased risk of cancer incidence and mortality of 2.7% and 9.6%, respectively.^1^ While the overall national cancer incidence has declined, a rural-urban disparity in cancer mortality has persisted for lung, prostate, breast, and colorectal cancers.^1,3^ Interest has increased in characterizing cancer disease burden between rural and urban areas across the cancer continuum; yet, most of these observations focus solely on cancer incidence and mortality outcomes and not survivorship. As a population-based indicator, survival rates report the elapsed time to death and illuminate advancements in and access to screening, treatment technologies, and quality clinical care.^4^ Cancer survival rates should be understood in the context of cancer burden. However, reports on potential rural-urban disparities in survival rates remain a gap in the literature. To date, no studies have reported on the trends in survival for cancer types that have higher rates of incidence and mortality for rural residents.

Geographic location alone does not predict cancer risk or survival. Rather, certain sociodemographic factors associated with higher cancer risk (eg, poorer socioeconomic conditions, reduced access to health care facilities, and racial segregation) are often overrepresented in rural communities.^1,5,6,7,8,9,10^ Moreover, largely because of systematic marginalization (eg, historical segregation, and immigration laws), higher cancer incidence and mortality are observed in racially and ethnically minoritized groups, and it is important to understand survival trends among these groups. Collectively, these and other factors are associated with delayed cancer detection, more severe disease outcomes, and increased cancer mortality. These factors are also associated with poorer survivorship outcomes.^5,6,11^ However, trends in rural-urban differences in sociodemographic and health care factors and their association with the survival rate of lung, prostate, breast, and colorectal cancer types are unknown.

This study examines rural-urban trends in cancer survival using data from the Surveillance, Epidemiology, and End Results (SEER) of patients with cancer diagnosed from 1975 to 2011. We expand on previous research that reports elevated incidence and mortality rates of lung, prostate, breast, and colorectal cancer in rural areas,^1,8,9,10,12,13,14^ with a detailed examination of rural-urban trends in 5-year cancer-specific survival probability (hereafter referred to as 5-year survival) in lung, prostate, breast, and colorectal cancer by race.

## Methods

This cross-sectional study followed the Strengthening the Reporting of Observational Studies in Epidemiology (STROBE) reporting guideline. We used the Surveillance Epidemiology and End Results 18 (SEER) from 1975 to 2016 to obtain potential participants. The National Cancer Institute sponsors the SEER program, which collects cancer data from population-based cancer registries that capture approximately 34.6% of the US population.^15^ The SEER data set contains deidentified data; therefore, this study is exempt from a full board review by the institutional review boards at the participating research institutions.

### Study Participants

We included patients diagnosed with lung, prostate, breast, and colon or rectal (henceforth, colorectal) cancer types with malignant behavior between 1975 and 2011, and we defined cancers using the *International Classification of Diseases for Oncology, Version 3* (*ICD-O-3*). We limited the sample to patients diagnosed until 2011 to allow for 5 years of follow-up time. We originally identified 1 190 724 lung cancer cases, 997 685 colorectal cancer cases, 1 385 576 female breast cancer cases, and 1 307 833 male prostate cancer cases. We excluded patients who were 18 years or younger, had missing information regarding their geocodes (ie, rural or urban designation), and had missing follow-up time. The final sample had 888 338 patients with lung cancer, 750 704 patients with colorectal cancer, 987 826 patients with breast cancer, and 1 032 549 patients with prostate cancer.

### Outcome, Exposure, and Covariates

The primary outcome was the 5-year cancer-specific survival—the estimated likelihood of death for the average cancer patient accounting for age, race and ethnicity, year, urbanicity, and tumor stage up to 60 months following diagnosis. To classify the urban or rural patients, we used the 2013 Rural-Urban Continuum Codes (RUCC), which classify metropolitan counties by population size and nonmetropolitan counties by the degree of urbanization and their proximity to a metropolitan area.^16^ Consistent with previous rural-urban thresholds,^17,18,19^ we classified patients in counties with codes 1 to 3 as urban, while all counties with codes 4 to 9 were rural. Covariates that were known risk factors for cancer survival included patient-level characteristics (ie, age, race and ethnicity, year of the diagnosis) and tumor characteristics (ie, tumor stage).

SEER-coded race and ethnicity data were obtained through electronic medical records, clinician notes, photographs, and any other sources used to determine race. Race and ethnicity categories included Asian and Pacific Islander, Hispanic, non-Hispanic Black, and non-Hispanic White. Tumor stage was classified as localized, regional, and distant based on SEER historic staging adjustments. Prostate cancer only had staging diagnosis for localized and distant for all years. We did not use the American Joint Committee on Cancer tumor, nodes, and metastases (TNM) staging system for tumor stage because the TNM staging criteria were not consistent during the study period.

### Statistical Analysis

We compared the distribution of the SEER sample characteristics between rural and urban residence overall and by cancer types. We presented these data as the median (IQR) for nonnormal continuous variables and column relative frequencies (proportions) for categorical variables. We compared the survival function of each cancer type and rurality by race and rurality using the Kaplan-Meier method. After confirming the proportionality of hazards assumptions, we estimated the 5-year survival by fitting a Cox proportional hazards regression model to account for rural-urban residence, age, race, year, and tumor stage. We also assessed the multiplicative interaction between race and rurality on each cancer outcome in our Cox proportional hazard models and examined the Wald χ^2^ (all interaction terms were statistically significant, *P* < .001). We censored patients at the time of their cancer-specific death or at 60 months of follow-up, excluding participants diagnosed after 2012 because they could not observe up to 60 months of follow-up time. We then used a generalized linear regression model (with a Poisson distribution) to estimate the mean probability of 5-year survival for each stratum of interest based on the probabilities observed from our Cox proportional hazards model. We used SAS statistical version 9.4 (SAS Institute) for these analyses. Finally, we used the Joinpoint Regression Program, version 4.5.0.1 (National Cancer Institute) to estimate the periods of significant increases or decreases in survival via joinpoint regression models. The joinpoint regression models tested which trends (between join points) were statistically significant and then estimated the annual percentage change (APC) in survival based on the generalized linear model between the 2 join point years. We present models stratified by rural or urban residence at diagnosis in the primary analysis, while secondary analyses include further stratification by race or ethnicity. We used a significance level of *P* < .05, and all tests were 2-tailed.

## Results

Using SEER data among cases diagnosed from 1975 to 2011, there were 3 659 417 cancer cases diagnosed, including cancers of the lung (888 338 [24.3%]), breast (987 826 [27.0%]), prostate (1 023 549 [28.0%]), and colorectal (750 704 [20.5%]) (Table). The median (IQR) age was 67 (58-76) years, and 1 918 609 (52.4%) were male patients. Overall, 237 815 patients (6.5%) were Hispanic, 396 790 (10.8%) were Black, and 2 825 037 (77.2%) were White, 1 750 535 of 3 659 417 (47.8%) were diagnosed with localized tumors, and 2 155 467 of 3 659 417 (58.9%) were diagnosed after age 65. Additionally, 2 304 978 of 3 659 417 patients with cancer (63.0%) were diagnosed between 2000 and 2011. Rural vs urban patients with cancer were more likely to be non-Hispanic White individuals (398 661 of 430 353 [92.6%] vs 2 664 191 of 3 229 064 [82.5%]) and aged 75 years or older (134 765 of 430 353 [31.3%] vs 916 256 of 3 229 064 [28.4%]). The overall 5-year survival rates increased from 1975 to 2011 for all cancer types (eFigure 1 in the Supplement). The rural 5-year survival rates were often lower than urban 5-year survival rates for all cancer types (eFigure 2 in the Supplement). Yet, the rural and urban 5-year survival rates were equivalent for most cancer types toward the end of the observed period; breast cancer had a persistent rural-urban 5-year survival gap throughout the entire observed period.

**Table.** Summary of Population Characteristics of Cancer Site Diagnosis Stratified by Urban or Rural Residence, Surveillance, Epidemiology, and End Results Data Among Patients Diagnosed Years 1975 to 2011

Characteristic	Patient, No. (%)										
	Total (N = 3 659 417)			Lung (N = 888 338)		Breast (N = 987 826)		Prostate (N = 1 032 549)		Colorectal (N = 750 704)	
	Overall (n = 3 659 417)	Urban (n = 3 229 064)	Rural (n = 430 353)	Urban (n = 772 561)	Rural (n = 115 777)	Urban (n = 886 035)	Rural (n = 101 791)	Urban (n = 915 763)	Rural (n = 116 786)	Urban (n = 654 705)	Rural (n = 95 999)
Median age (IQR), years	67 (58-76)	67 (58-76)	69 (60-77)	69 (61-77)	69 (61-76)	61 (50-72)	64 (53-74)	68 (61-75)	70 (63-76)	70 (60-79)	71 (61-79)
Male, ^a^	1 918 609 (52.4)	1 681 456 (52.1)	237 153 (55.1)	434 970 (56.3)	71 415 (61.7)	886 035 (0)	101 791 (0)	915 763 (100)	116 786 (100)	330 723 (50.5)	48 952 (51.0)
Female	1 740 808(47.6)	1 547 608(47.9)	193 200 (44.9)	337 591 (43.7)	44 362 (38.3)	886 035 (100)	101 791 (100)	0	0	323 982 (49.5)	47 047 (49.0)
Race											
Hispanic (all races)	237 815 (6.5)	224 128 (6.9)	13 687 (3.2)	35 870 (4.6)	2572 (2.2)	71 631 (8.1)	3664 (3.6)	69 132 (7.6)	4359 (3.7)	47 495 (7.3)	3092 (7.3)
Non-Hispanic Asian	170 162 (4.6)	165 671 (5.1)	4491 (1.0)	35 989 (4.7)	1056 (0.9)	52 745 (6.0)	1312 (1.3)	37 617 (4.1)	1024 (0.9)	39 320 (6.0)	1099 (6.0)
Non-Hispanic Black	396 790 (10.8)	373 273 (11.6)	23 517 (5.5)	88 831 (11.5)	5795 (5.0)	86 931 (9.8)	5012 (4.9)	126 978 (13.9)	7935 (6.8)	70 533 (10.8)	4775 (5.0)
Non-Hispanic White	2 825 037 (77.2)	2 440 063 (75.6)	384 974 (89.5)	609 273 (78.9)	105 779 (91.4)	669 220 (75.5)	90 800 (89.2)	668 108 (73.0)	102 214 (87.5)	493 462 (75.4)	86 181 (89.8)
Other^b^	29 613 (0.8)	25 929 (0.8)	3684 (0.9)	2598 (0.3)	575 (0.5)	5508 (0.6)	1003 (1.0)	13 928 (1.5)	1254 (1.1)	3895 (0.6)	852 (0.6)
Tumor Site^c^											
Localized	1 750 535 (47.8)	1 557 075 (48.2)	193 460 (45.0)	119 182 (15.4)	17 795 (15.4)	530 304 (59.9)	60 828 (59.8)	652 185 (71.2)	77 702 (66.5)	255 404 (39.0)	37 135 (38.7)
Regional	753 584 (20.6)	664 406 (20.6)	89 178 (20.7)	155 996 (20.2)	24 561 (21.2)	276 094 (31.2)	30 754 (30.2)	NA	NA	232 316 (35.5)	33 863 (35.3)
Distant	618 405 (16.9)	540 184 (16.7)	78 221 (18.2)	316 101 (42.2)	48 402 (41.8)	55 634 (6.3)	6834 (6.7)	31 176 (3.4)	4401 (3.8)	127 273 (19.4)	18 584 (19.4)
Unknown	536 893 (14.7)	467 399 (14.5)	69 494 (16.2)	171 282 (22.2)	25 019 (21.6)	24 003 (2.7)	3375 (3.3)	232 402 (25.4)	34 683 (29.7)	39 712 (6.1)	6417 (6.7)
Age of diagnosis											
≤44	187 462 (5.1)	171 671 (5.3)	15 791 (3.7)	19 058 (2.5)	2100 (1.8)	117 016 (13.2)	9968 (9.8)	4531 (0.5)	310 (0.3)	31 066 (4.8)	3413 (3.6)
45-54	456 577 (12.5)	412 163 (12.8)	44 414 (10.3)	74 341 (9.6)	10 445 (9.0)	193 930 (21.9)	18 433 (18.1)	70 162 (7.7)	6309 (5.4)	73 730 (11.3)	9227 (9.6)
55-64	859 911 (23.5)	763 717 (23.7)	96 194 (22.4)	176 559 (22.9)	27 254 (23.5)	208 260 (23.5)	23 808 (23.4)	250 323 (27.3)	26 897 (23.0)	128 575 (19.6)	18 235 (19.0)
65-74	1 104 446 (30.2)	965 257 (29.9)	139 189 (32.3)	254 238 (32.9)	40 669 (35.1)	187 195 (21.1)	24 630 (24.2)	347 500 (38.0)	46 467 (39.8)	176 324 (26.9)	27 423 (28.6)
≥75	1 051 021 (28.7)	916 256 (28.4)	134 765 (31.3)	248 365 (32.2)	35 309 (30.5)	179 634 (20.3)	24 952 (24.5)	243 247 (26.6)	36 803 (31.5)	245 010 (37.4)	37 701 (39.3)
Year of diagnosis											
1975-1979	166 851 (4.6)	144 008 (4.5)	22 843 (5.3)	39 535 (5.1)	5218 (4.5)	39 160 (4.4)	5711 (5.6)	24 927 (2.7)	4834 (4.1)	40 386 (6.2)	7080 (7.4)
1980-1984	196 853 (5.4)	170 216 (5.3)	26 637 (6.2)	48 505 (6.3)	6573 (5.7)	44 925 (5.1)	6227 (6.1)	31 237 (3.4)	5947 (5.1)	45 549 (7.0)	7890 (8.2)
1985-1989	233 943 (6.4)	203 137 (6.3)	30 806 (7.2)	54 338 (7.0)	7415 (6.4)	56 931 (6.4)	7658 (7.5)	42 774 (4.7)	7451 (6.4)	49 094 (7.5)	8282 (8.6)
1990-1994	351 126 (9.6)	314 035 (9.7)	37 091 (8.6)	73 280 (9.5)	8313 (7.2)	80 157 (9.1)	8450 (8.3)	98 764 (10.8)	11 983 (10.3)	61 834 (9.4)	8 345 (8.7)
1995 -1999	405 666 (11.1)	367 176 (11.4)	38 490 (8.9)	84 591 (11.0)	9051 (7.8)	102 853 (11.6)	9608 (9.4)	106 708 (11.7)	10 984 (9.4)	73 024 (11.2)	8847 (9.2)
2000-2004	946 648 (25.9)	833 689 (25.8)	112 959 (26.3)	194 753 (25.2)	32 046 (27.7)	227 437 (25.7)	26 449 (26.0)	247 476 (27.0)	30 637 (26.2)	164 023 (25.1)	23 827 (24.8)
2005-2009	966 714 (26.4)	851 428 (26.4)	115 286 (27.8)	198 890 (25.7)	33 583 (29.0)	234 935 (26.5)	26 614 (26.2)	258 442 (28.2)	32 213 (27.6)	159 161 (24.3)	22 876 (23.8)
2010-2011	391 616 (10.7)	345 375 (10.7)	46 241 (10.7)	78 669 (10.2)	13 578 (11.7)	99 637 (11.3)	11 074 (10.9)	105 435 (11.5)	12 737 (10.9)	61 634 (9.4)	8852 (9.2)

Abbreviation: SEER, Surveillance, Epidemiology, and End Results.

^a^ For sex-specific cancers of breast and prostate, we only included women and men respectively.

^b^ Other race and ethnicity includes American Indian, Alaskan Native, and unknown race or ethnicity.

^c^ For the SEER historical variable of tumor stage, prostate cancer did not have “regional” diagnoses characterized separately.

### Rural and Urban 5-Year Lung Cancer Survival Rates by Racial and Ethnic Groups 

**Figure 1.**
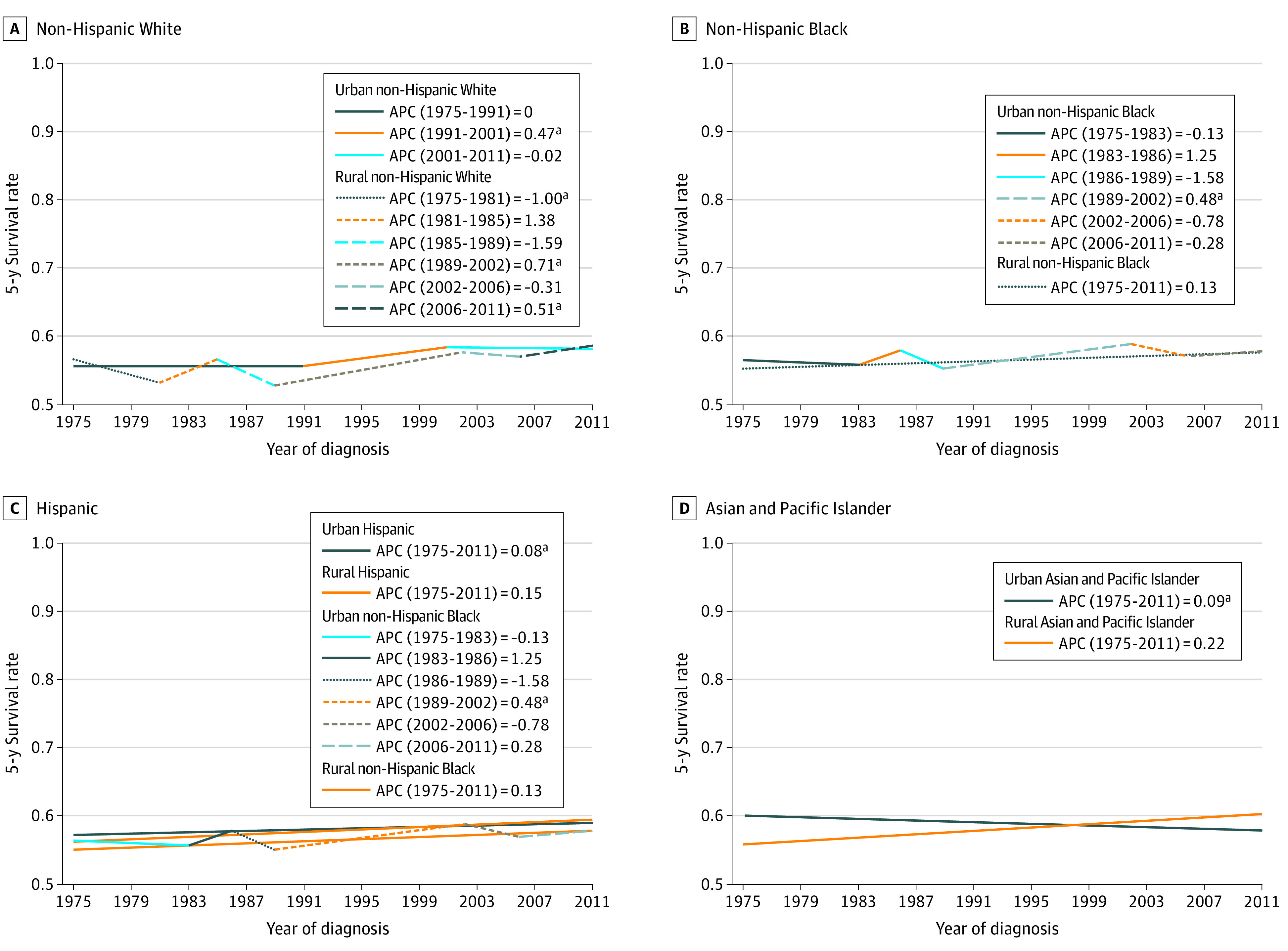
Annual Percent Change (APC) in Rural and Urban 5-Year Survival Rates for Lung Cancer by Racial and Ethnic Groups ^a^*P* < .05.

Panel A in Figure 1 shows the 5-year lung cancer survival rate for urban non-Hispanic White patients increased from 57.7% to 58.3%, with a single period of significant increase in survival from 1991 to 2001 (55.7%-58.4%; 95% CI, 0.31-0.63; *P* < .001). During the observed period, the 5-year survival rate for rural non-Hispanic White patients increased from 56.6% to 58.6%, with 3 periods of significant change from 1975 to 1981 (56.9%-53.3%; 95% CI, −1.86 to −0.12; *P* < .03); 1989 to 2002 (52.8%-57.9%; 95% CI, 0.51 to 0.90; *P* < .001); and 2006 to 2011 (57.2%-58.6%; 95% CI, 0.04 to 0.98; *P* < .04). Panel B in Figure 1 shows that the 5-year lung cancer survival rate of urban non-Hispanic Black patients increased from 56.4% to 57.8%, with a single period of significant increase from 1989 to 2002 (55.2%-58.8%; 95% CI, 0.34 to 0.63; *P* < .001). For rural non-Hispanic Black patients, the survival rate increased from 55.1% to 57.7%, with no significant change and the lowest 5-year survival rate among all races and cancer types. Panel C in Figure 1 shows that the 5-year lung cancer survival rate significantly increased during a single period for urban Hispanic patients from 57.2% to 59.0% (95% CI, 0.01 to 0.16; *P* < .03), while the rural Hispanic 5-year survival rate was not significant. Panel D in Figure 1 shows that the urban and rural 5-year lung cancer survival had a uniquely inverse association among Asian and Pacific Islander patients. The 5-year survival rate of urban Asian and Pacific Islander patients significantly decreased from 60.1% to 58.1% (95% CI, −0.16 to −0.02; *P* < .01), while rural 5-year survival rate was not significant.

### Rural and Urban 5-Year Survival Rates for Prostate Cancer by Racial and Ethnic Groups 

**Figure 2.**
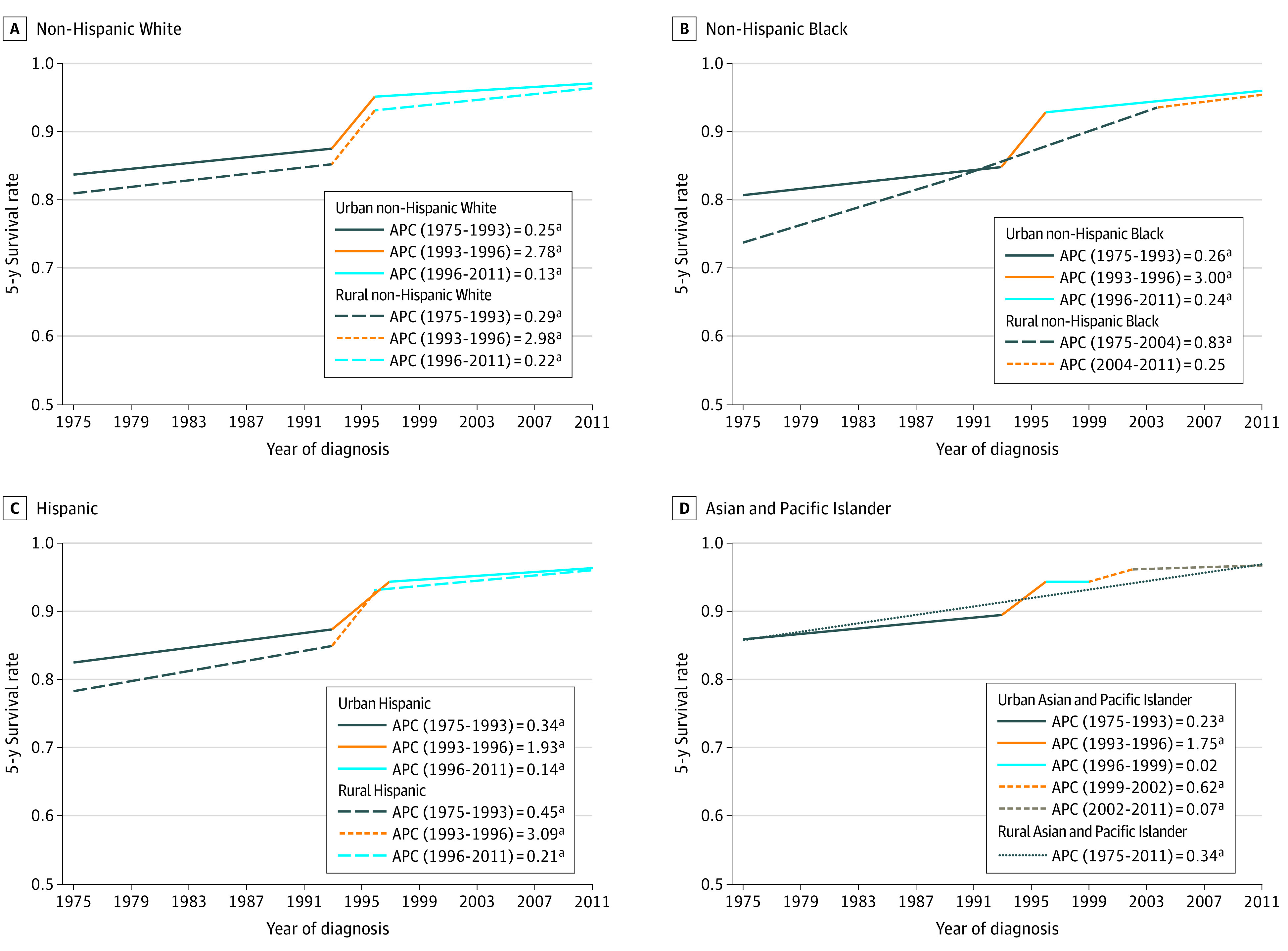
Annual Percent Change (APC) in Rural and Urban 5-Year Survival Rates for Prostate Cancer by Racial and Ethnic Groups ^a^*P* < .05.

Panel A in Figure 2 shows that urban non-Hispanic White men with prostate cancer had 3 periods of consecutive significant survival rate increases from 1975 to 1993 (83.7% to 87.6%; 95% CI, 0.22 to 0.28; *P* < .001), to 1996 (92.5%; 95% CI, 2.19 to 3.37; *P* < .001), and 2011 (96.9%; 95% CI, 0.11 to 0.15; *P* < .001). There were also 3 periods of consecutive significant survival rate increases for rural non-Hispanic White men with prostate cancer from 1975 to 1993 (81.1% to 85.5%; 95% CI, 0.24 to 0.34; *P* < .001), to 1996 (90.6%; 95% CI, 1.74 to 4.23; *P* < .001), and 2011 (96.5%; 95% CI, 0.19 to 0.26; *P* < .001). Panel B of Figure 2 shows that the 5-year prostate cancer survival rate for urban non-Hispanic Black men had 3 consecutive periods of significant increases from 1975 to 1993 (80.9% to 84.8%; 95% CI, 0.20 to 0.32; *P* < .001), to 1996 (92.7%; 95% CI, 1.88 to 4.13; *P* < .001), and 2011 (96.0%; 95% CI, 0.20 to 0.27; *P* < .001). The 5-year prostate cancer survival rate for rural non-Hispanic Black men increased from 73.8% to 95.4% during the observed period, with a single period of significant increase from 1975 to 2004 (73.8% to 93.8%; 95% CI, 0.67 to 0.99; *P* < .001). Panel C in Figure 2 shows that the 5-year prostate cancer survival rate for urban Hispanic men had 3 significant increases from 1975 to 1993 (82.3% to 87.4%; 95% CI, 0.21 to 0.46; *P* < .001), to 1997 (92.6%; 95% CI, 1.13 to 2.73; *P* < .001), and 2011 (96.2%; 95% CI, 0.10 to 0.19; *P* < .001). For rural Hispanic men with prostate cancer, there were 3 consecutive periods of significant increase in survival from 1975 to 1993 (78.4% to 85.0%; 95% CI, 0.31 to 0.60; *P* < .001), to 1996 (93.1%; 95% CI, 0.03 to 6.25; *P* < .05), and 2011 (96.1%; 95% CI, 0.10 to 0.32; *P* < .001). Panel D in Figure 2 depicts the 5-year prostate cancer survival rate for urban Asian and Pacific Islander men with 4 consecutive periods of increase from 1975 to 1993 (85.8% to 89.5%; 95% CI, 0.15 to 0.31; *P* < .001), to 1996 (94.3%; 95% CI, 0.83 to 2.68; *P* < .001), 1999 to 2002 (94.3% to 96.1%; 95% CI, 0.09 to 1.16; *P* < .02), and 2011 (96.7%; 95% CI, 0.03 to 0.12; *P* < .03). For rural Asian and Pacific Island men, there was a single period of significant increase in prostate cancer survival from 1975 to 2011 (86.3% to 96.9%; 95% CI, 0.18 to 0.51; *P* < .001).

### Rural and Urban 5-Year Breast Cancer Survival Rates by Racial and Ethnic Groups 

**Figure 3.**
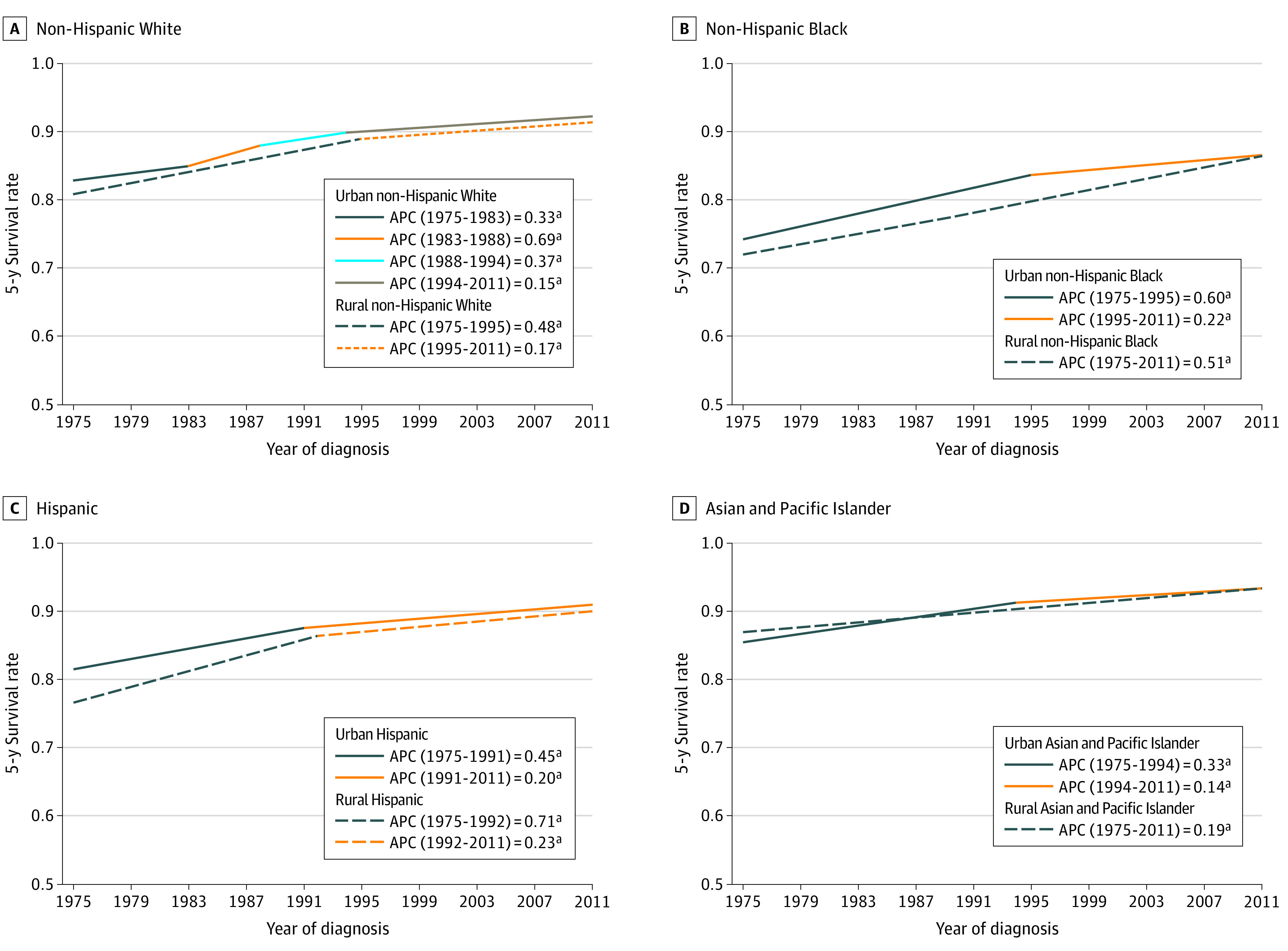
Annual Percent Change (APC) in Rural and Urban 5-Year Survival Rates for Breast Cancer by Racial and Ethnic Groups ^a^*P* < .05.

Panel A in Figure 3 depicts 4 consecutive periods of significant increase in the breast cancer survival rate for urban non-Hispanic White women from 1975 to 1983 (82.9%-85.1%; 95% CI, 0.26-0.39; *P* < .001), to 1988 (88.0%; 95% CI, 0.52-0.85; *P* < .001), to 1994 (90.0%; 95% CI, 0.27-0.47; *P* < .001), and 2011 (92.4%; 95% CI, 0.14-0.16; *P* < .001). There were 2 consecutive periods of significant increase in the 5-year breast cancer survival rate for rural non-Hispanic White women beginning in 1975 to 1995 (80.9%-89.1%; 95% CI, 0.44-0.53; *P* < .001) and 2011 (91.5%; 95% CI, 0.13-0.21; *P* < .001). Panel B in Figure 3 reports 2 consecutive significant increases in the breast cancer survival rate for urban non-Hispanic Black women from 1975 to 1995 (74.2%-83.7%; 95% CI, 0.54-0.66; *P* < .001) and 2011 (86.7%; 85% CI, 0.18-0.27; *P* < .001). For non-Hispanic Black rural women, the 5-year breast cancer survival rate significantly increased from 1975 to 2011 (72.0%-86.5%; 95% CI, 0.37-0.65; *P* < .001). Panel C in Figure 3 details that the 5-year breast cancer survival rate for urban Hispanic women increased from 1975 to 1991 (81.5%-88.1%; 95% CI, 0.33-0.56; *P* < .001) and 2011 (91.1%; 95% CI, 0.17-0.22; *P* < .001). For rural Hispanic women, 5-year breast cancer survival rate significanly increased during 2 consecutive periods from 1975 to 1992 (76.7%-86.5%; 95% CI, 0.47-0.95; *P* < .001) and 2011 (90.4%; 95% CI, 0.12-0.35; *P* < .001). Panel D in Figure 3 shows the 5-year breast cancer survival rate for urban Asian and Pacific Islander women had 2 consecutive periods of significant increase from 1975 to 1994 (85.7%-91.2%; 95% CI, 0.26-0.39; *P* < .001) and 2011 (93.4%; 95% CI, 0.12-0.16; *P* < .001). For rural Asian and Pacific Islander women, the 5-year survival probability increased from 87.3% to 93.4% (95% CI, 0.09-0.29; *P* < .001).

### Trends in Rural and Urban 5-Year Colorectal Cancer Survival Rates for Racial and Ethnic Groups 

**Figure 4.**
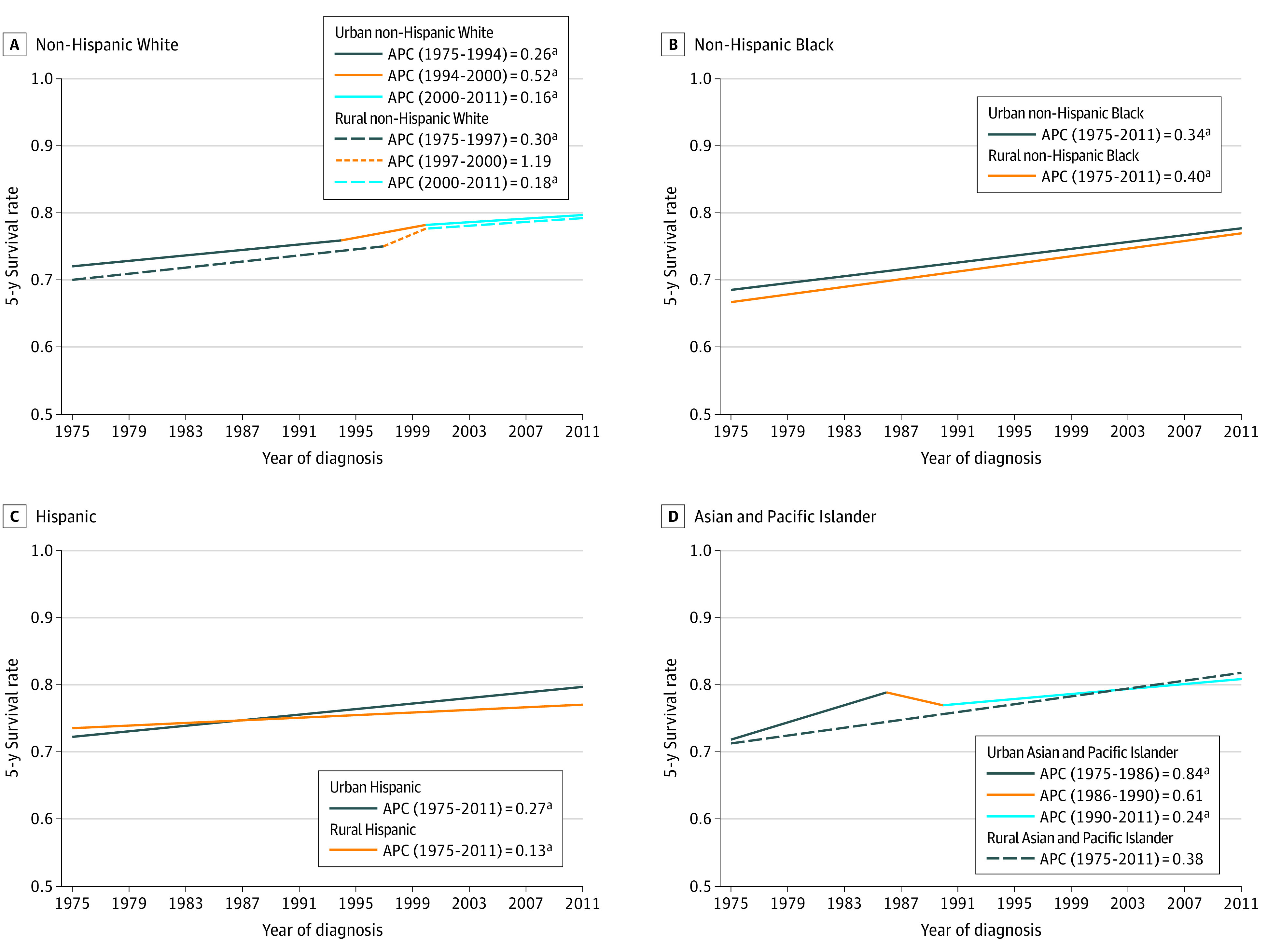
Annual Percent Change (APC) in Rural and Urban 5-Year Survival Rates for Breast Cancer by Racial and Ethnic Groups ^a^*P* < .05.

Panel A in Figure 4 shows that the 5-year colorectal cancer survival rate for urban non-Hispanic White patients had 3 consecutive periods of increase during the observed period, beginning 1975 to 1994 (72.2%-75.7%; 95% CI, 0.22-0.29; *P* < .001), to 2000 (78.3%; 95% CI, 0.29-0.76; *P* < .001), to 2011 (79.7%; 95% CI, 0.11-0.21; *P* < .001). The 5-year survival rate for rural non-Hispanic White patients with colorectal cancer had 2 periods of significant increase from 1975 to 1997 (69.6%-74.6%; 95% CI, 0.23-0.37; *P* < .001) and from 2000 to 2011 (77.9%-79.9%; 95% CI, 0.07-0.29; *P* < .002). Panel B in Figure 4 shows that the 5-year colorectal cancer survival rate for urban and rural non-Hispanic Black patients had a single increase during entire observed period of 65.6% to 77.5% (95% CI, 0.31-0.37; *P* < .001) and 71.3% to 75.6% (95% CI, 0.25-0.55; *P* < .001), respectively. Similarly, Panel C in Figure 4 shows that both urban and rural Hispanic patients with colorectal cancer had a single increase during the entire observed period of 70.0% to 79.3% (95% CI, 0.23-0.31; *P* < .001) and 74.5% to 77.3% (95% CI, 0.01-0.24; *P* < .03), respectively. Panel D in Figure 4 shows that the colorectal cancer survival rate of urban Asian and Pacific Islander patients had 2 periods of significant increase from 1975 to 1986 (71.6%-78.9%; 95% CI, 0.47-1.20; *P* < .001) and from 1991 to 2011 (76.6%-81.1%; 95% CI, 0.20-0.28; *P* < .001). The 5-year colorectal survival rate increase for rural Asian and Pacific Islander patients was not significant.

## Discussion

To our knowledge, this is the first study to examine the 5-year survival by rural-urban and racial or ethnic categories for the 4 cancer types with the highest incidence and mortality rate in rural areas—lung, prostate, breast, and colorectal cancers. From 1975 to 2011, the overall rural-urban 5-year survival rate increased substantially across these cancer types. We examined these rural-urban trends by race and ethnicity. Generally, the 5-year survival of non-Hispanic Black and rural patients was consistently lower than urban patients for each cancer type, independent of sociodemographic or health care variables. Our findings measured the annual percent change, which can be compared with periods of marked advancements in detection, treatments, and access to treatments that contributed to increases in survival.^8,10,20^ Our study builds on the mounting evidence of poorer survivorship outcomes for rural patients and fills an important knowledge gap with a descriptive report of rural-urban 5-year survival probability trends that have persisted for almost 40 years.

During the observed period, cancer survival trends narrowed between urban and rural patients for all racial and ethnic groups. Although non-Hispanic White patients, generally, had the highest 5-year cancer survival rates in urban and rural categories, our findings emphasize persistent health inequities in cancer survivorship for rural and racially and ethnically minoritized groups. Disparities affecting racial and ethnic cancer survival rates are partly explained by evidence of systemic inequities that lend to racial and ethnic minoritized groups experiencing lower levels of health literacy, income, and access to health care coverage in the United States, all of which are associated with increased cancer-specific mortality.^21,22,23^ These findings punctuate the need for elevated health care resources and education for racially and ethnically minoritized groups, especially those in rural settings. While the source of these disparities is multifactorial, health equity interventions, programs, and policies are warranted to eliminate disparities.

The observed disparities in rural-urban survival are largely influenced by disparities present upstream in the cancer care continuum. Multiple studies report rural-urban disparities in access to screening facilities, cancer education programming, treatment facilities, and navigation services.^8,9,10,20,24,25^ Targeted programming for rural cancer survival will have tempered effects without targeted programming focusing on rural care across the cancer continuum. Yet, funding through the National Cancer Institute R-series grants addressing rural cancer control and management remains low.^1^ Furthermore, funded grants focused solely on rural populations, rather than rural-urban differences, are even less. Although existing funding facilitates limited growth in rural cancer research, policy reform targeting rural cancer control remains minimal. In 2020, the American Society of Clinical Oncology released a policy statement that identified that persistent inequities and rural cancer burden support the development and advancement of programs that improve financing for cancer care and interventions that improve access to care.^26^ Additional research, programming, financial resources, and policy changes are needed to comprehensively address rural-urban cancer disparities along the cancer continuum.

This study has several strengths, including that this is the first comprehensive decades-long descriptive study of rural-urban 5-year survival across multiple common cancer types in rural areas. We intend to generate hypotheses related to the comprehensive observation of rural-urban disparities trend over time, thus informing future investigations and policies. To supplement this data, future studies that explore the role of both modifiable factors (access to care^11,27,28^ [eg, broadband internet, treatment modalities] and health behaviors [eg, sun exposure, smoking behaviors]^17,29^), and nonmodifiable factors (socioeconomic status^30,31,32^ [eg, poverty, occupation]) in survival trends are warranted.

Another strength is that we used a large nationally representative data set observing 36 years of urban and rural cancer survival trends, which were stratified by key demographic and clinical factors. This investigation aims to illuminate long-term rural-urban and racial and ethnic survival trends. These long-term trends serve to characterize cancer burden since the development of modern technology and treatment advancements.

### Limitations

This study had limitations. We used 2013 RUCCs to classify rural and urban areas as early as 1975. Notably, rural areas have experienced sporadic population growth since 1970.^33^ Therefore, our findings are limited by misclassification bias as a subsample of counties that were classified as urban in this study but truly classified as rural in 1975. These potential misclassifications will likely bias the results toward the null but have minimal impact on the aim of the study, which provides a descriptive analysis of the observed trends. We limited our data analysis to SEER 18 registries and included cases diagnosed until 2011 because subsequent SEER data sets (ie, SEER 2019 and SEER 2020) only included data as early as 2000, rather than 1975.

## Conclusions

In this study, the rural 5-year survival probabilities for lung, prostate, breast, and colorectal cancers were consistently lower than urban survival probabilities from 1975 to 2011. Although the probability of survival increased during the observed time, rarely did the differences between urban and rural patients narrow. Policy changes, research, and financial supports are urgently needed to further investigate and close the 36-year gap that persists in urban and rural probabilities of cancer survival.

## Notes

Supplement. **eFigure 1.** Annual Percent Change (APC) in Overall 5-Year Survival Rates by Cancer Type **eFigure 2.** Annual Percent Change (APC) in Rural and Urban 5-Year Survival Rates by Cancer Type
