# Errors in Author Contributions and Abstract

**DOI:** 10.1001/jamanetworkopen.2022.21167

**Published:** 2022-06-28

In the Original Investigation titled “Racial and Ethnic Differences in Rural-Urban Trends in 5-Year Survival of Patients With Lung, Prostate, Breast, and Colorectal Cancers: 1975-2011 Surveillance, Epidemiology, and End Results (SEER),”^1^ published May 19, 2022, there was an extra word in the Abstract and a misspelling in Dr Langston’s name in the Author Contribution section. This article has been corrected.^1^
