# Supplementary material for: Racial and Ethnic Differences in Rural-Urban Trends in 5-Year Survival of Patients With Lung, Prostate, Breast, and Colorectal Cancers: 1975-2011 Surveillance, Epidemiology, and End Results (SEER)
Source: JAMA Netw Open. 2022 May 19;5(5):e2212246. doi: 10.1001/jamanetworkopen.2022.12246 (PMC9121191; doi:10.1001/jamanetworkopen.2022.12246)
Supplement: Supplement. — eFigure 1. Annual Percent Change (APC) in Overall 5-Year Survival Rates by Cancer Type eFigure 2. Annual Percent Change (APC) in Rural and Urban 5-Year Survival Rates by Cancer Type [file jamanetwopen-e2212246-s001.pdf]

Supplemental Online Content

Lewis-Thames MW, Langston ME, Khan S, et al. Racial and ethnic differences in rural-urban trends in 5-year survival of patients with lung, prostate, breast, and colorectal cancers: 1975-2011 Surveillance, Epidemiology, and End Results (SEER). *JAMA Netw Open*. 2022;5(5):e2212246. doi:10.1001/jamanetworkopen.2022.12246

eFigure 1. Annual Percent Change (APC) in Overall 5-Year Survival Rates by Cancer Type
eFigure 2. Annual Percent Change (APC) in Rural and Urban 5-Year Survival Rates by Cancer Type

This supplemental material has been provided by the authors to give readers additional information about their work.

© 2022 Lewis-Thames MW et al. *JAMA Network Open.*

eFigure 1. Annual Percent Change (APC) in Overall 5-Year Survival Rates by Cancer Type

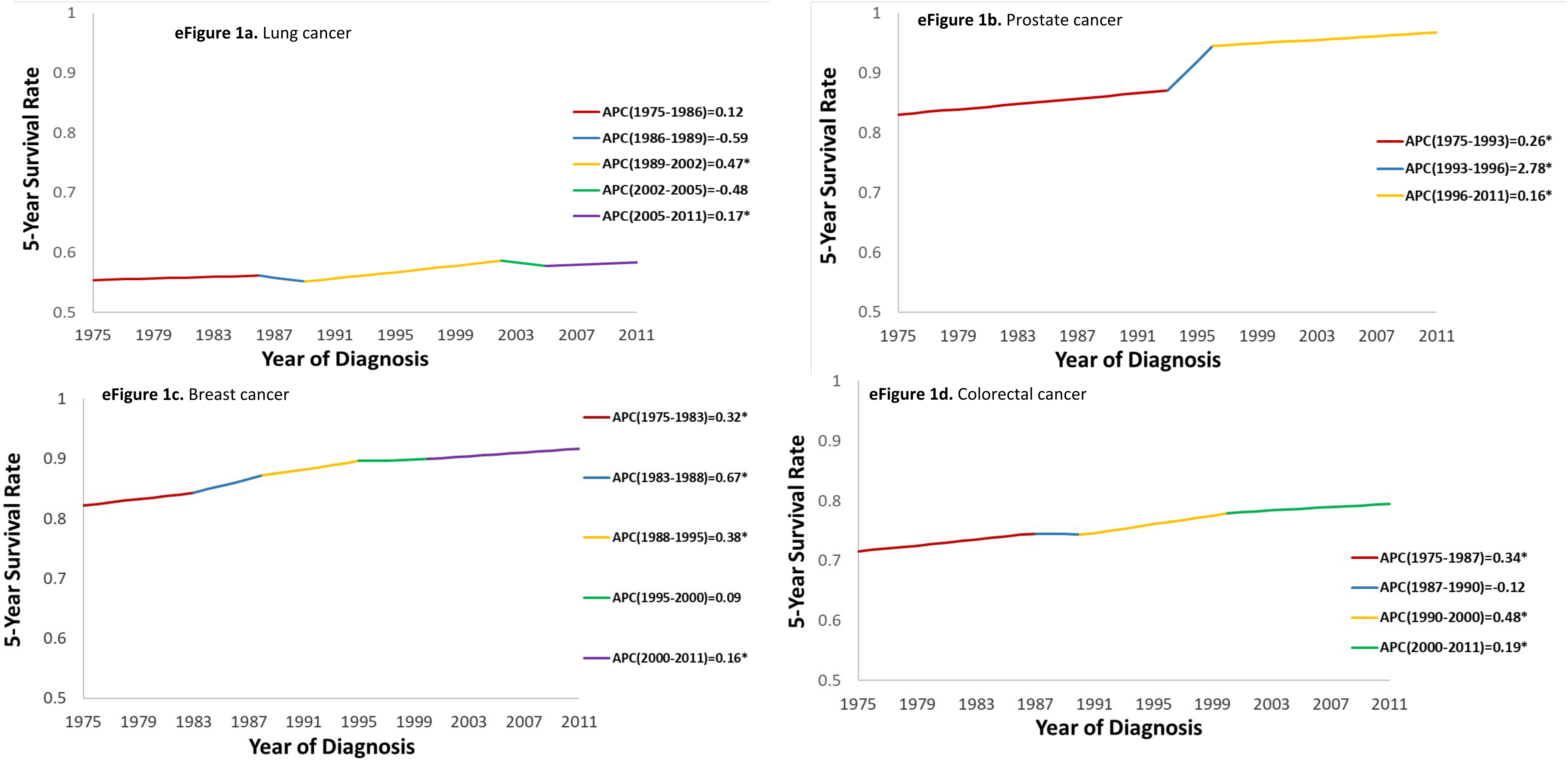

© 2022 Lewis-Thames MW et al. *JAMA Network Open.*

eFigure 2. Annual Percent Change (APC) in Rural and Urban 5-Year Survival Rates by Cancer Type

eFigure 2a. Lung cancer

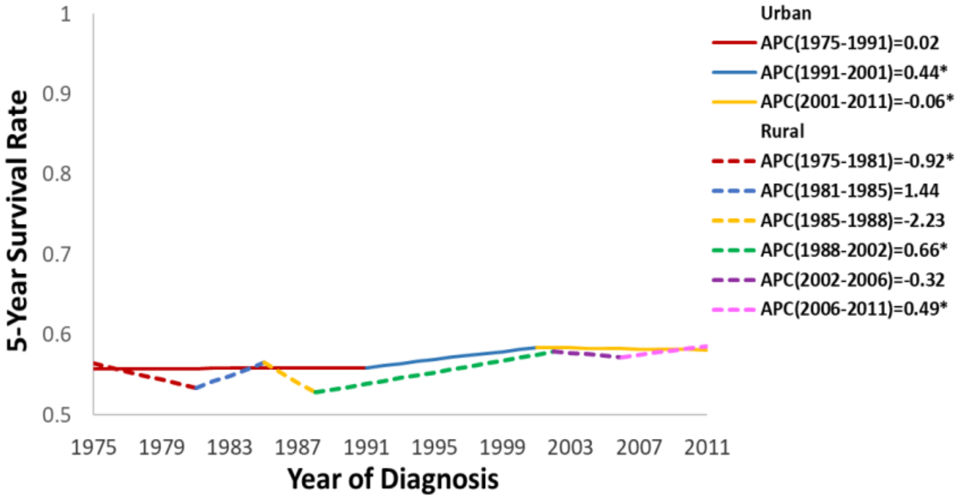

eFigure 2b. Prostate cancer

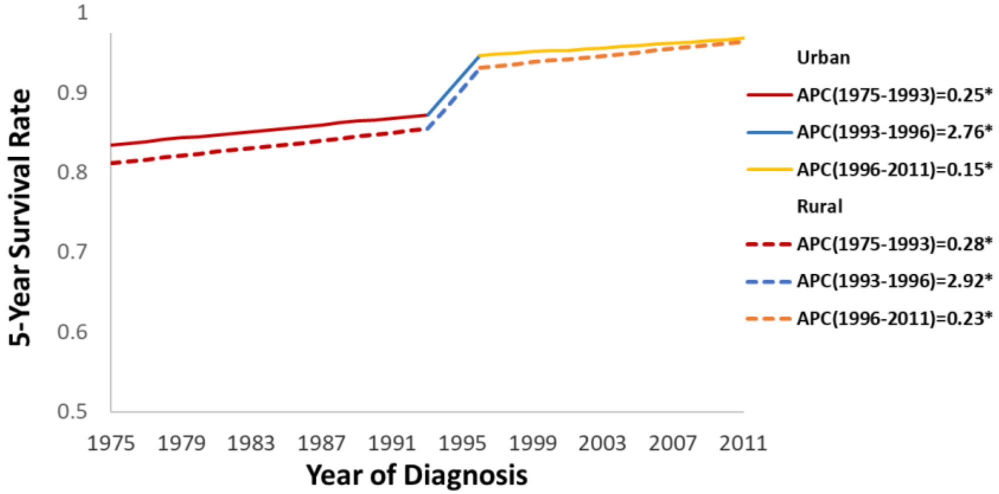

eFigure 2c. Breast cancer

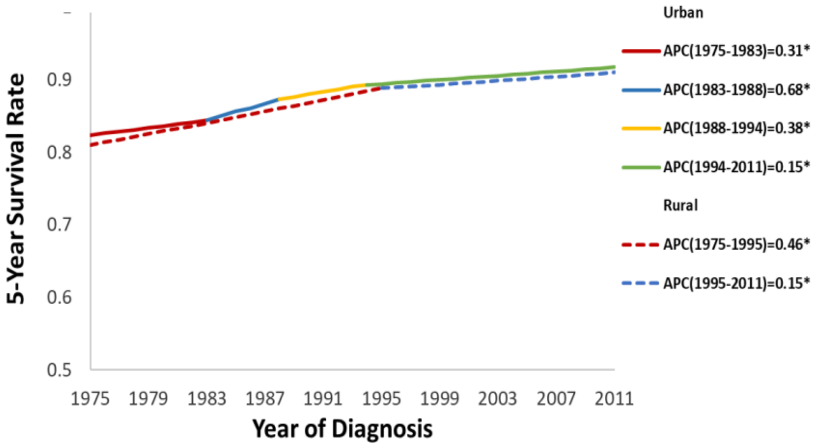

eFigure 2d. Colorectal cancer

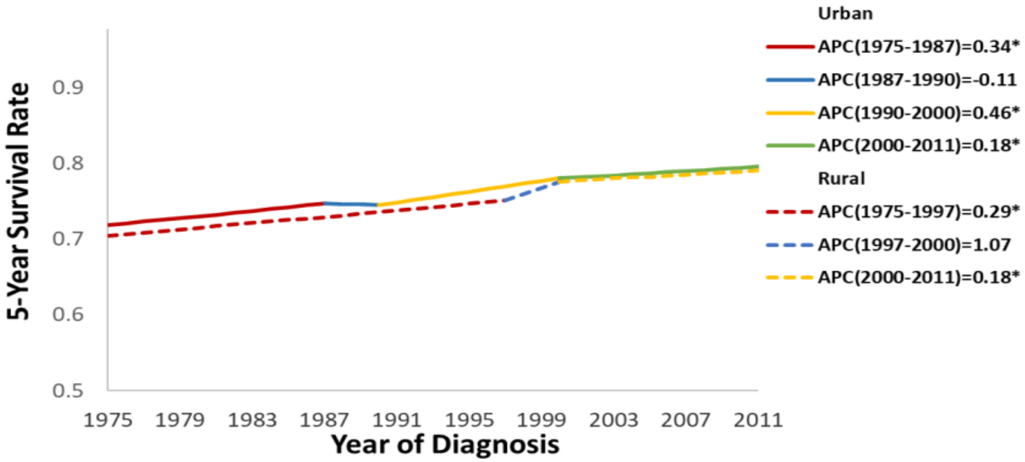

© 2022 Lewis-Thames MW et al. *JAMA Network Open.*
